# Hijacking the Ubl code: bacterial manipulation of ubiquitin-like proteins

**DOI:** 10.1042/EBC20253059

**Published:** 2025-12-08

**Authors:** Shun-Je Bhark, Rachel E. Lacoursiere, Jonathan N. Pruneda

**Affiliations:** 1 Department of Molecular Microbiology and Immunology, Oregon Health & Science University, Portland, OR, 97239, U.S.A.; 2 Department of Chemical Physiology and Biochemistry, Oregon Health & Science University, Portland, OR, 97239, U.S.A.; 3 Knight Cancer Institute, Oregon Health & Science University, Portland, OR, 97239, U.S.A.

**Keywords:** bacterial pathogens, effector, ubiquitin-like conjugation, Ubl

## Abstract

Ubiquitin (Ub) and Ub-like (Ubl) signaling processes regulate broad aspects of eukaryotic cellular biology. Conserved sets of enzymes control the covalent attachment of Ub/Ubl onto proteins, and disruption of these highly regulated processes contributes to diseases including cancer and neurodegeneration. Aspects of Ub/Ubl signaling are central to the innate immune response to infectious pathogens. As such, pathogens such as viruses and bacteria have evolved sophisticated mechanisms to hijack and dysregulate the homeostasis of Ub/Ubl signaling. Pathogenic manipulation of the host Ub system is well studied, with multiple classes of secreted bacterial effector proteins discovered that regulate either Ub itself or the enzymes required for substrate ubiquitylation. While much less is known about the control of host Ubl signaling processes by pathogens, recent discoveries indicate that they, too, are hijacked during infection. The number of Ubl manipulators secreted by bacterial pathogens is likely to increase in the coming years as methods to identify and characterize bacterial effectors advance. This review highlights the current knowledge on bacterial manipulation of Ubl signaling, including SUMO, NEDD8, ISG15, UFM1, FAT10, and LC3.

## Introduction

**Figure 1:**
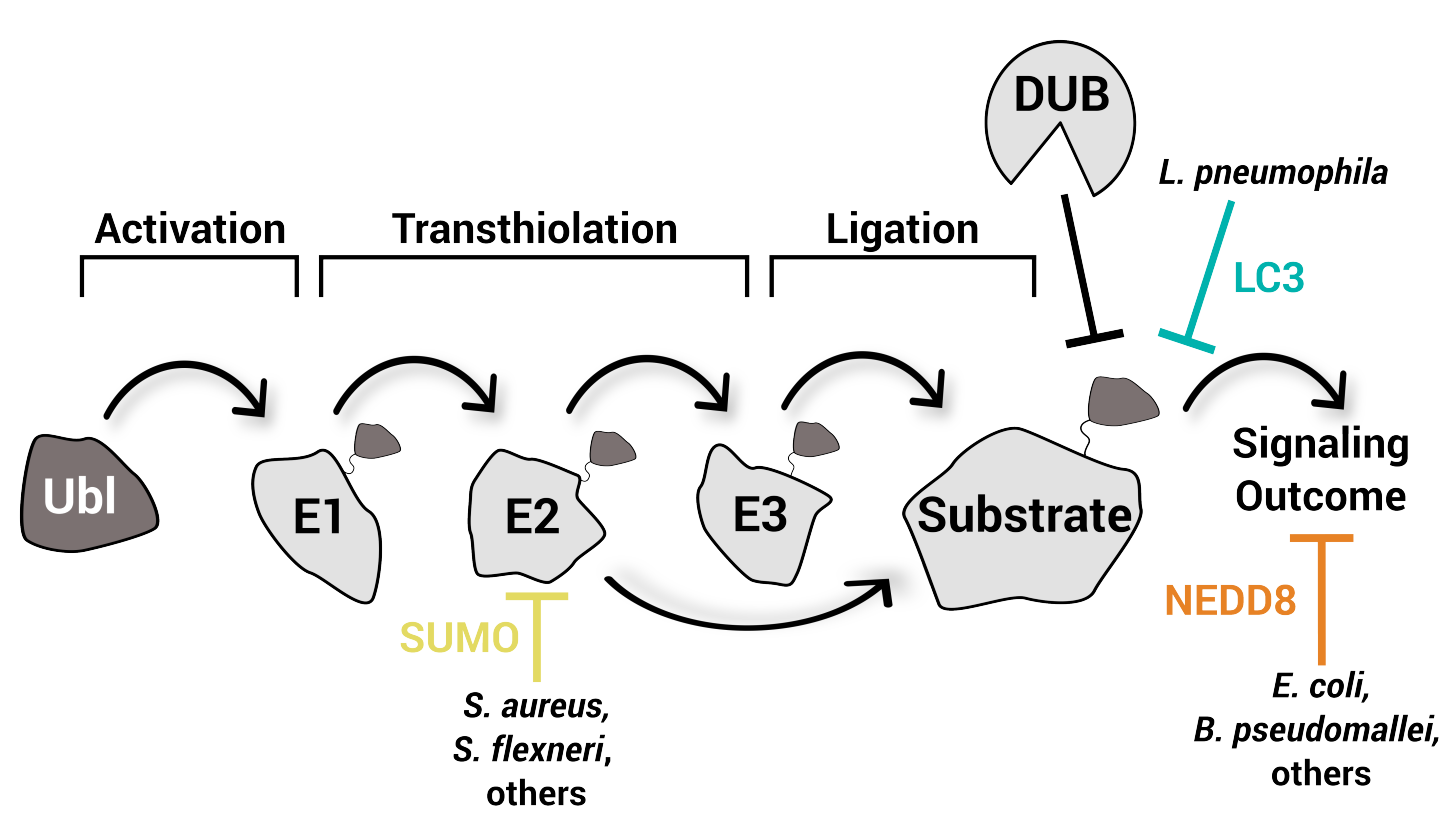
Various steps in Ubl signaling are targeted by bacteria. Canonical Ubl signaling follows an E1, E2, E3 cascade that facilitates the covalent modification of a substrate with the Ubl. The main steps required are 1) activation of the Ubl; 2) transthiolation between enzymes; 3) ligation onto a substrate; and 4) the signaling outcome. Pathogenic bacteria demonstrated to interfere with specific parts of the cascade are highlighted in the figure along with their corresponding Ubl. To date, no bacterial effector proteins have been shown to redirect Ubl ligation through ligase activity.

The conjugation of ubiquitin (Ub) or ubiquitin-like proteins (Ubls) has vast regulatory roles in cells including proteostasis, innate and adaptive immunity, and transcription/translation [1–6]. Ub and Ubl signaling follow similar regulatory cascades, whereby an E1-activating enzyme first adenylates the Ub/Ubl C-terminus and forms an activated thioester intermediate through a catalytic cysteine, prior to transfer onto the catalytic cysteine of an E2-conjugating enzyme. An E2~Ub or E2~Ubl conjugate can then coordinate with specific E3 ligases to covalently modify substrates, thereby facilitating downstream signaling through specific Ub/Ubl-binding proteins. Deconjugation of Ub/Ubl from the substrate occurs through the activity of deubiquitylases or Ubl proteases, which regenerate the pool of unconjugated Ub/Ubl (Figure 1). Moving through the cascade, the number of enzymes classified in each category increases: While this process can be massively complex, as is evident by the ~1000 proteins that regulate ubiquitin signaling in humans, other Ubl processes are minimalistic by comparison and consist of only a few enzymes known to regulate each step. Specific enzymes involved in the cascade of each Ubl are discussed below in the corresponding sections.

**Figure 2:**
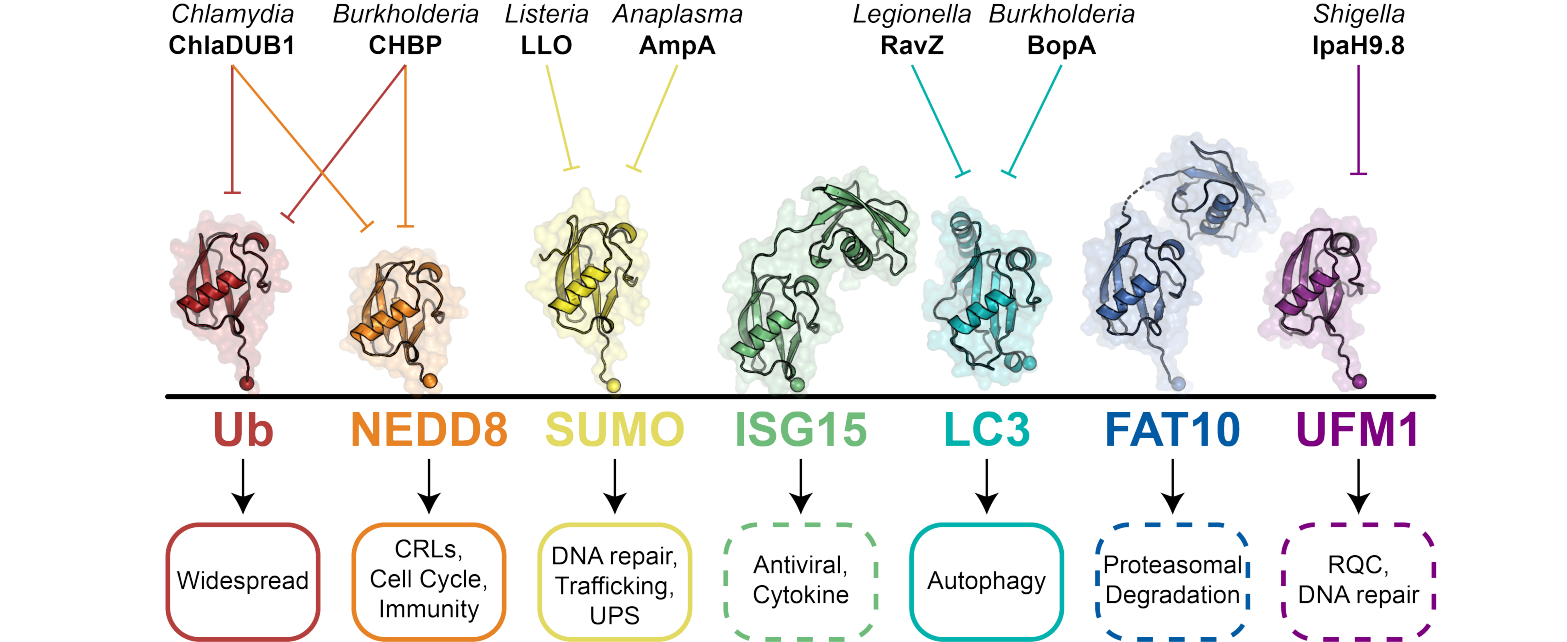
Pathogenic bacteria hijack the Ubl code. Three dimensional structures of Ub and Ubl modifiers illustrate conservation of the beta grasp fold and variation in N-terminal extensions (Ub, PDB 1UBQ; NEDD8, PDB 1NDD; SUMO1, PDB 1Y8R; ISG15, PDB 7S6P; LC3B, PDB 5GMV; FAT10, PDB 7PYV; UFM1, PDB 5L95). Simplified representations of Ub/Ubl signaling pathways are depicted below, with dashed lines encircling those with roles that continue to emerge (CRLs, Cullin RING Ligases; UPS, Ubiquitin-Proteasome System; RQC, Ribosomal Quality Control). Above, selected examples of bacterial interference in Ub/Ubl signaling are shown, with inhibitory functions represented by blunted arrows.

Although ubiquitylation and Ub have been extensively studied, the discovery and study of Ubl pathways is ongoing. The small ubiquitin-like modifier (SUMO) family (consisting of SUMO1, SUMO2, and SUMO3), neural precursor cell expressed developmentally down-regulated protein-8 (NEDD8), interferon-induced 15 kDa protein (ISG15), ubiquitin-fold modifier (UFM1), microtubule-associated protein 1 light chain 3 (LC3), and human leukocyte antigen-F adjacent transcript 10 (FAT10) all resemble and behave similarly to Ub through their conjugation to substrate proteins, with the exception of LC3 which is conjugated to the lipid phosphatidylethanolamine. Sequence and structural similarity vary between all Ubls, with NEDD8 being the most similar to Ub. While most are made up of a minimal beta-grasp fold, some Ubl proteins include short N-terminal extensions (e.g., SUMO1 or LC3), or even a second beta-grasp domain (e.g., ISG15 or FAT10). Aside from FAT10, all Ub/Ubl proteins require proteolytic post-translational processing of their C-termini. In the case of Ub, deubiquitylases remove either ribosomal subunits or additional copies of Ub, revealing the C-terminal diglycine motif required for conjugation. Ubl proteases process the C-termini of their respective Ubl proteins by removing short peptides, revealing either C-terminal diglycine (e.g., ISG15, NEDD8, or SUMO) or glycine (e.g., LC3 or UFM1) motifs. In addition to these differences in the structure and processing of the Ub/Ubl itself, the signaling outcomes associated with substrate modification vary widely depending on the Ub/Ubl (Figure 2). Among many great reviews, readers are invited to read references [2–6].

Among its many functions, the ubiquitylation system plays key roles in pathogen recognition during infection, for example, by forming a signaling platform surrounding cytosolic bacteria required for the recruitment of autophagy receptors such as OPTN, NDP52, and p62 [7]. To counter these early defense mechanisms, many pathogens have evolved sophisticated mechanisms to subvert the host ubiquitylation system including encapsulation, establishing a vacuolar niche, or deploying virulence-associated effectors into the host cytosol [8]. Both viral and bacterial pathogens utilize effector proteins; hereafter referred to as effectors, to promote their virulence, and many have been shown to modulate host ubiquitylation. Pathogenic strategies to manipulate ubiquitylation, reviewed extensively elsewhere [9], can include 1) alteration of the Ub surface (as in *Chromobacterium violaceum* CteC or *Legionella pneumophila* SdeA); 2) alteration of ubiquitylation enzymes (as in *Shigella flexneri* OspG or *Legionella pneumophila* MavC); or 3) modulation of ubiquitin signals (as in *Burkholderia pseudomallei* TssM or *Chlamydia trachomatis* ChlaDUB1/2) [10–16]. Recent advancements have also highlighted a growing number of bacterial effectors that target Ubls and their conjugation (Figures 1 and 2). In this review, we will discuss these Ubl-targeting effectors and how they are deployed during infection.

## NEDD8

NEDD8 is the most similar Ubl to Ub, sharing 58% sequence identity. The NEDD8-activating enzyme consists of two subunits, NAE1 and UBA3, which together form a heterodimer that structurally resembles the Ub E1-activating enzyme UBA1. NAE1/UBA3 distinguishes NEDD8 from Ub by recognizing Ala72 in NEDD8, which is Arg72 in Ub [17]. Compared with Ub, which conjugates to ~35 of the E2 enzymes, NEDD8 conjugates to only UBE2F or UBE2M. The primary role for NEDDylation is arguably the allosteric control of cullin RING Ub ligases (CRLs), which require NEDD8 modification within the cullin subunit for activation [18]. Other roles for NEDDylation may exist downstream of the IAPs and RNF35/TRIM40 E3 ligases, both of which are also active Ub ligases [19].

Due to its regulation of CRLs, NEDDylation is involved in many cellular processes including cell cycle regulation and immune signaling [20,21]. These functions make the NEDDylation pathway an ideal target for pathogens to manipulate during infection. While more is known about the roles of NEDDylation during viral infection [22], a number of bacteria secrete effector proteins that modulate NEDDylation. The Cif (cycle inhibiting factor) family of proteins has homologues across many bacterial pathogens including *Escherichia coli*, *Yersinia pseudotuberculosis*, *Burkholderia pseudomallei* (named CHBP), and *Photorhabdus luminescens*. Cif proteins arrest cells at G1/S or G2/M transitions during the cell cycle through impaired activity of CRLs and failed clearance of cell cycle regulators [23–25]. Cif proteins bind NEDD8 to promote deamidation of Gln40, converting the residue to glutamate [26–30]. While the conversion of Gln40 to Glu40 in NEDD8 does not impair NEDDylation of cullin proteins, the charged Glu40 residue impacts the allosteric activation of CRL complexes [26]. Unsurprisingly, Cif proteins also deamidate the conserved Gln40 residue in Ub, though Cifs from the species mentioned above display a preference for NEDD8, likely due to its stronger binding affinity [26–29].

Further regulation of NEDDylation can be observed by the deubiquitylases RickCE (*Rickettsia bellii*), ChlaDUB1, and ChlaDUB2 (*Chlamydia trachomatis*) [16,31]. Although these enzymes preferentially liberate Ub from conjugated proteins, they retain activity toward NEDD8 conjugates as well due to its similarity to Ub. Analogous to the human NEDD8 protease, NEDP1, ChlaDUB1 can process the immature pro-NEDD8 and cleave model NEDDylated substrates but cannot remove NEDD8 from cullin [31]. ChlaDUB1/2 were discovered during mammalian cell infection by *C. trachomatis* [16] and at least ChlaDUB1 was determined to be a late effector due to its abundance at 16 h and 24 h post infection [32]. ChlaDUB1 is reported to interfere with programmed cell death, inflammatory NF-κB signaling, nutrient availability, and Golgi fragmentation [32–35]; however, whether these roles are tied to the DUB or deNEDDylase function of ChlaDUB1 is unclear. Interestingly, ChlaDUB1 also exhibits acetyltransferase activity through the same catalytic cysteine [35]. In this case, structure-guided mutations have enabled studies that assign its proteolytic and acetylation activities to biological functions [35,36]. Similar separation-of-function mutations for DUB and deNEDDylase activities of ChlaDUB1 could be highly valuable for future work. Likewise, the role of RickCE in *R. bellii* infection has not been explored and could uncover interesting roles of NEDD8 manipulation at the host–pathogen interface.

## SUMO

SUMOylation follows a similar conjugation cascade to Ub; however, only a single E1 (consisting of a dimer of SAE1/SAE2) and a single E2 (UBE2I) have been identified. Deconjugation of SUMO is accomplished by six SUMO-specific proteases (SENPs). Additionally, three SUMO isoforms in humans can take part in SUMOylation, each with distinct, but overlapping target substrates. Human SUMO2 and SUMO3 share 95% sequence identity and are indistinguishable by commercial antibodies; as such, they are commonly referred to as SUMO2/3. SUMO1 shares 46% and 48% sequence identity with SUMO2 and SUMO3, respectively. Owing to sequence differences, SUMO1 can only be utilized in monoSUMOylation reactions or as a cap to polySUMO chains, whereas SUMO2/3 can be assembled into polySUMO chains formed primarily via Lys11 located in an unstructured extension N-terminal to the conserved beta-grasp fold. SUMOylation controls important cellular processes such as nuclear-cytosolic transport, protein stability, and transcriptional regulation. Thus, it is not surprising that bacterial pathogens have evolved mechanisms to hijack this important signal.

While there is strong evidence of direct SUMO manipulation among plant pathogens, likely due to its regulation of stress responses [37], to date, most identified mechanisms for bacterial manipulation of the human SUMOylation system involve indirect effects on the expression of SUMO regulators. *Listeria monocytogenes* [38], *Staphylococcus aureus* [39], *Staphylococcus warneri* [40], *Shigella flexneri* [41], and *Salmonella* Typhimurium [42] have been shown to reduce the abundance of UBE2I. Most targeting of UBE2I appears to be through an indirect mechanism, with data indicating that *L. monocytogenes* and *S. warneri* rely on a pore-forming effector. How perturbations to membrane integrity result in decreased abundance of UBE2I remains to be elucidated. *S*. Typhimurium is an exception, as Verma et al. have shown that infection upregulates the mature form of host miR30 microRNAs that reduce expression of UBE2I [42]. UBE2I is not the only target of bacterial manipulation, as *S. flexneri* and *S. warneri* have been shown to also reduce the abundance of SAE1 [43] and SAE1/SAE2 [40], respectively.

Despite the lack of a clear mechanism in most cases, the fact that multiple pathogens appear to reduce the abundance of UBE2I indicates that SUMOylation may play an important role in coordinating antibacterial responses. However, alternate evidence shows that some effectors either up-regulate SUMOylation or must be SUMOylated themselves to function, highlighting the complex interplay between activation or inhibition of SUMOylation that occurs during bacterial infection. For example, despite evidence showing that *S. flexneri* decreases SUMOylation by reducing the abundance of UBE2I, the effector OspF must be SUMOylated in order to be trafficked into the nucleus where it can accomplish its function of disrupting transcription [44]. Other bacterial species, such as *Anaplasma phagocytophilum,* an understudied intracellular pathogen, also appear to require SUMOylation to achieve optimal infection. *A. phagocytophilum* secretes AmpA during initial infection and replication [45]. Beyer et al. have shown that AmpA localizes to the exterior of the vacuole that *A. phagocytophilum* persists in and that AmpA is polySUMOylated. When SUMOylation is globally inhibited with a SAE1/SAE2 inhibitor, bacterial load is considerably reduced. Supporting this, Truchan et al. have shown that recruitment of the intermediate fiber vimentin to the *Anaplasma*-containing vacuole requires SUMOylation and that vimentin recruitment may be necessary for optimal bacterial growth [46]. Finally, Dunphy et al. show that the obligate intracellular pathogen *Ehrlichia chaffeensis* requires SUMOylation of the secreted effector TRP120 for optimal replication [47]. SUMOylated TRP120 recruits several host proteins to the *Ehrlichia*-containing vacuole, and inhibition of SAE1/SAE2 abrogates this activity, resulting in reduced bacterial load.

That pathogens appear capable of utilizing and/or suppressing the SUMOylation pathway highlights both the important role that SUMOylation plays in key cellular functions and the complexity of SUMO signaling. While there are not yet any examples of direct SUMO manipulation among human pathogens, growing evidence suggests that they likely exist and await future discovery.

## ISG15

As its name suggests, ISG15 and many of its regulatory enzymes are transcriptionally controlled by interferon signaling. Expression of the E1 (UBE1L), E2 (UBE2L6), E3 (HERC5), and deconjugating protease (USP18) are all regulated alongside ISG15 expression [48]. ISG15 and its regulatory enzymes have been studied much more intensively for their involvement in antiviral defense. To counter these functions, viruses have adopted multiple mechanisms of ISG15 interference, including viral proteases that act as deISGylases. These viral effectors have been reviewed previously [49], and we will instead focus on bacterial manipulations of this Ubl.

To date, there are no identified instances of bacterial effectors that directly target the ISGylation system, in part due to the difficulty of studying this Ubl. Unlike ubiquitin, ISG15 is capable of functioning as an extracellular cytokine in addition to functioning as a post-translational modification [50]. Additionally, trypsin digestion of ISGylated proteins forms the same diglycine remnants as ubiquitin and NEDD8, which exacerbates the difficulty of confirming putative substrates. Finally, a signaling role for ISGylation is still heavily debated. While some models present a co-translational role in modifying newly synthesized proteins during infection [51], others propose a role in transient, noncovalent interactions that are essential for the activation of certain proteins [52]. However, these difficulties do not preclude the possibility of discovering bacterial ISG15-targeting effectors. Several studies already point to the important antibacterial role of ISG15. For example, Swaim et al. have shown that human peripheral blood mononuclear cells (PBMCs) exposed to heat-inactivated *Mycobacterium bovis* BCG, *Mycobacterium tuberculosis*, or *Salmonella* Typhimurium in concert with IL-12 can produce and secrete a robust level of ISG15 [53]. These findings agree with additional data that demonstrate the importance of ISG15 in restricting both *Mycobacterial* infections [54], as well as *Listeria monocytogenes* infections [55–57]. In these contexts, roles for ISG15 include restricting bacterial infection by inducing autophagy [56] or preventing the formation of actin tails required for motility [57]. Given these findings, it is likely that bacterial pathogens have evolved effectors to hijack these important antibacterial roles of ISG15. It is also possible that pathogenic bacteria may seek to enhance ISGylation. Although this would be contradictory given the findings previously described, Wu et al. have demonstrated that ISG15 signaling functions as an immune ‘brake’ during infection with *Chlamydia trachomatis*. In experiments with ISG15-knockout mice, Wu et al. demonstrated greater inflammation and delayed bacterial clearance [58]. Similar immunomodulatory effects of ISG15 are seen during infection with *M. tuberculosis*. Kimmey et al. observed immune-activating and immune-suppressing roles for ISG15 dependent on the duration of infection [59]. Taken together, these findings support a role for ISG15 in functioning as a modulator of the immune response, which represents an enticing target for bacterial pathogens who would greatly benefit from exploiting this function.

Although no direct manipulators of ISG15 have been discovered in bacteria, an effector that may indirectly manipulate ISGylation has been described in *Streptococcus pneumoniae*. Cao et al. have demonstrated a potential role for the secreted effector LytA in manipulating host ISGylation [60]. LytA is a secreted autolysin that uniquely hydrolyzes the cell wall of *S. pneumoniae* without affecting other bacterial species. Normally, detection of *S. pneumoniae* DNA induces a robust interferon response, with up-regulation of ISG15 and its regulatory machinery. However, the presence of LytA results in less accumulation of *S. pneumoniae* DNA in the cytosol of macrophages, resulting in a less robust up-regulation of ISGylation. It is unclear whether this is a result of direct manipulation of ISG15 signaling or the result of a general decrease in interferon signaling, as Cao et al. also report an increase in abundance of the ISG15 protease, USP18, in cells exposed to *S. pneumoniae* DNA. USP18 binds to the Type I interferon receptor and blocks JAK1 activation of interferon signaling, which could also explain the reduction in ISGylation [61]. Although there is clearly still much to learn about ISG15 manipulation by bacteria, the results reported by Cao et al. are perhaps the first step in identifying additional bacteria and specific effectors that can manipulate ISGylation.

## LC3 (ATG8)

The LC3 subfamily of Ubls, consisting of LC3A, LC3B, and LC3C, are mammalian homologs of yeast Atg8 and play an essential role in development and elongation of the autophagosome membrane. LC3 undergoes a cleavage and activation cascade similar to other Ubls that ultimately results in its conjugation onto phosphatidylethanolamine (PE) at the surface of the maturing phagosome. Following maturation to reveal the C-terminal glycine motif, LC3 is activated by the E1-like ATG7, transferred to the E2-like ATG3, and finally conjugated onto PE. LC3 plays a key role in selective autophagy, whereby it serves as an interaction platform for LC3-interacting region (LIR)-containing autophagy adaptor proteins that, in turn, can recruit macromolecular structures such as damaged organelles or invading bacteria for destruction [62]. The targeted autophagic degradation of intracellular bacteria, termed xenophagy, forms an important arm of cell-autonomous immunity [63]. As a result, the LC3 signaling pathway has attracted several examples of bacterial manipulation.

The respiratory pathogen *Legionella pneumophila* secretes the effector RavZ, a cysteine protease that cleaves LC3 from PE [64]. Remarkably, rather than cleaving after the C-terminal glycine like a traditional Ub/Ubl protease, RavZ cleaves one amino acid before to leave a glycine remnant behind on the PE. This ‘clippase’ specificity blocks the target from being modified again, and perhaps more importantly, prevents the released Ubl from being recycled through the conjugation cascade. Similar clippase activities have since been identified in other viral and bacterial Ub/Ubl proteases [65,66]. RavZ appears to be dispensable for intracellular growth in macrophages, as RavZ deletion has no impact on *L. pneumophila* growth. This is not unusual for *L. pneumophila* effectors, as single-effector mutants rarely have a phenotypic impact due to a high degree of functional redundancy among the >350 secreted effectors. However, RavZ homologs have been found in other species of *Legionella* [67], which supports its importance as many *Legionella* effectors are species-specific.

Other pathogens encode LIR-containing effector proteins, though their specific roles in LC3 manipulation during infection have yet to be fully elucidated. An *in silico* predictive screen of host protein and bacterial effector interactions identified a LIR within the *S*. Typhimurium effector YhjJ [68]. Subsequent infection studies in macrophages showed that YhjJ reduced accumulation of LC3 on the subpopulation of *S*. Typhimurium that escapes from the *Salmonella-*containing vacuole and persists in the cytosol. However, the specific function of YhjJ has yet to be determined. *Burkholderia pseudomallei* possesses two LIR-containing effectors, BopA and BPSL2203 [69,70]. *B. pseudomallei* persists in both the cytoplasm and within enclosed phagosomes in infected cells. BopA has been shown to be important for *B. pseudomallei* to break out of phagosomes and avoid autophagic degradation [71]. In the absence of BopA, LC3 accumulates at *Burkholderia-*containing vacuoles, suggesting that it plays a role in reducing detection and/or LC3 conjugation through an unknown mechanism. The LIR of BPSL2203 was discovered as part of a screen for LC3-interacting *B. pseudomallei* effectors using immunoaffinity chromatography [70]. However, no additional characterization of BPSL2203 or its role in manipulating LC3-mediated autophagy has been performed.

The apparently convergent nature of RavZ as an LC3 protease, coupled with the adaptation of LIR-containing effectors, indicates that bacteria use both familiar and unfamiliar methods to subvert LC3-mediated autophagy. While computational approaches have been successful in certain cases, unbiased approaches such as those used to identify RavZ and BPSL2203 will likely reveal novel strategies of autophagy disruption.

## FAT10

FAT10 has been shown to target proteins for proteasomal degradation [72,73] and has roles in regulating the ubiquitin system [74–76]. The conjugation cascade for FAT10 has not been fully elucidated. UBA6 and UBE2Z (USE1) are known to function as the E1 and E2, respectively, but no E3 ligase or proteases specific for FAT10 have been found. FAT10 is expressed in basal conditions only in immune cells, owing to its gene localization within that of the major histocompatibility complex class I locus [72]. However, FAT10 expression in nonimmune cells can be regulated through pro-inflammatory cytokines including interferon gamma (IFNɣ) or tumor-necrosis factor (TNF) [77]. As these pro-inflammatory cytokines are up-regulated during the host response to cellular infection, FAT10 would serve as an easy target for pathogen manipulation. To date, however, there have been no specific bacterial effectors identified that target FAT10. In an infection model, decoration of *S*. Typhimurium with FAT10 was associated with the activation of autophagy, and was observed as early as 1 h post infection [78]. Further, while cell-based experiments did not show an influence of FAT10 on *Salmonella* replication, an NRAMP1 transgenic mouse model demonstrated higher bacterial burden upon knockout of FAT10. In these experiments, FAT10 knockout also contributed to lower survival of the animals [78]. This work provides evidence that FAT10 is a useful antibacterial strategy that parallels other Ub or Ubl pathways, raising interest into whether bacteria have evolved methods to thwart these responses.

## UFM1

As is the case with many Ubl modifications, UFM1 is gaining appreciation for its involvement in a variety of cellular processes. During conjugation, UFM1 is initially activated by the E1 UBA5 prior to transfer onto the E2 UFC1. UFL1 functions as the E3 ligase for final transfer onto the substrate. Deconjugation of UFM1 from substrates is accomplished by UFSP1 and UFSP2. While UFMylation plays a prominent role as an essential responder in ribosome-associated quality control at the endoplasmic reticulum, many additional roles are being identified [79]. In recent work by Jing et al., UFMylation was shown to be essential for the proper regulation and activation of the inflammasome NLRP3 [80]. Inflammasomes function as immune sensors for danger signals such as bacterial antigens. Jing et al. demonstrated that UFMylation of NLRP3 prevents its ubiquitination and subsequent degradation. Using *E. coli-*induced sepsis in a mouse model, they showed that deficiency in the UFMylation system decreased the bacterial spread to the spleen and liver and reduced injury in the lung [80]. Conversely, multiple reports have identified UFMylation as a negative regulator for pro-inflammatory NF-κB and interferon signaling [81,82]. These roles stage UFMylation as a likely target for bacterial intervention. Although the mechanism is unclear, a genome-wide CRISPR screen identified the UFMylation pathway as a negative regulator for *L. pneumophila* infection in macrophages [83]. Likewise, a recent proximity biotinylation analysis identified a role for the UFMylation pathway in restricting intracellular *S. flexneri* and *S*. Typhimurium [84]. In the case of *S. flexneri*, the bacterial E3 ligase effector IpaH9.8 appears to protect the bacteria from decoration with UFM1, though the exact mechanism remains an open question.

## Conclusions

The future of Ubl research remains promising. Not only are Ubls becoming increasingly recognized for the complex variety of cellular processes they are involved in, but they are also becoming more recognized as important targets of pathogen manipulation. An abundance of literature has demonstrated roles for Ubls in viral infection, but bacterial manipulation of host Ubl signaling remains understudied. Specifically, much remains to be learned about how bacterial pathogens have evolutionarily acquired these effectors to hijack Ubl signaling. That the currently known effectors possess mechanisms that drastically differ from how their eukaryotic counterparts function suggests that these bacterial effectors have evolved independently, rather than having been acquired from eukaryotic hosts. However, much more mechanistic elucidation and discovery of additional Ubl-targeting effectors is necessary for a more complete evolutionary picture. The development of new tools and techniques to study Ubl regulation will greatly aid the discovery of novel Ubl-targeting effectors from pathogenic bacteria. Following success stories from the identification of Ub-targeting effectors, a combination of similarity-based and functional approaches could reveal additional modes of Ubl manipulation during bacterial infection [85,86]. Sequence and/or structural similarity searches, particularly now with the implementation of large-scale structural modeling, may lead to the identification of Ubl regulators in bacteria that exhibit similarity to known examples in eukaryotes. Alternatively, functional approaches to identify Ubl regulators would allow for unbiased identification of bacterial enzymes that may have arisen through convergent evolution. Ubl activity-based probes could be employed to capture cysteine-based enzymes that conjugate or hydrolyze Ubls, or the implementation of cross-linking or proximity labeling could reveal transient, noncovalent Ubl interactions. Finally, highly sensitive activity assays for Ubl conjugation or deconjugation may directly report on Ubl manipulation by secreted effectors. Thus, there is ample potential for discovery in exploring how bacteria hijack the Ubl code, and discoveries within this space hold the potential to inform on not only bacterial pathogenesis but on eukaryotic Ubl signaling as well.

**Summary Points**

- Pathogens manipulate ubiquitin and ubiquitin-like signaling to subvert host biology.
- Secreted bacterial effectors have been identified that directly or indirectly target Ubls, including SUMO, NEDD8, ISG15, and LC3.
- Other Ubls such as FAT10 and UFM1 interface with bacterial infection, though specific effectors that manipulate these processes have yet to be identified.
- Advancement of techniques to identify and characterize novel Ubl-modulating effectors will reveal new aspects of the host–pathogen interface.

## Open Access

This article has been published open access under our Subscribe to Open programme, made possible through the support of our subscribing institutions, learn more here: https://portlandpress.com/pages/open_access_options_and_prices#conditional

## Abbreviations

FAT10: human leukocyte antigen-F adjacent transcript 10
ISG15: interferon-stimulated gene 15 kDa protein
LC3: Microtubule-Associated Protein 1 Light Chain 3
NEDD8: Neural precursor cell expressed developmentally down-regulated protein 8
SUMO: small ubiquitin-like modifier
UFM1: ubiquitin-fold modifier 1
Ub: ubiquitin
Ubl: ubiquitin-like

## References

[1] Komander D. Rape M 2012 The ubiquitin code Annu. Rev. Biochem. 81 203 229 10.1146/annurev-biochem-060310-170328 22524316

[2] Schmidtke G. Aichem A. Groettrup M 2014 FAT10ylation as a signal for proteasomal degradation Biochim. Biophys. Acta 1843 97 102 10.1016/j.bbamcr.2013.01.009 23333871

[3] Lee B. Jeong H. Kim Y. Roh J.S. Sohn D.H 2025 Ubiquitin-like proteins in autoimmune diseases: current evidence and therapeutic opportunities Immune Netw. 25 e21 10.4110/in.2025.25.e21 40620421 PMC12226256

[4] Karpiyevich M. Artavanis-Tsakonas K 2020 Ubiquitin-Like Modifiers: Emerging Regulators of Protozoan Parasites Biomolecules 10 1403 10.3390/biom10101403 33022940 PMC7600729

[5] Kang J.A. Kim Y.J. Jeon Y.J 2022 The diverse repertoire of ISG15: more intricate than initially thought Exp. Mol. Med. 54 1779 1792 10.1038/s12276-022-00872-3 36319753 PMC9722776

[6] K. S.T. Joshi G. Arya P. Mahajan V. Chaturvedi A. Mishra R.K 2021 SUMO and SUMOylation pathway at the forefront of host immune response Front. Cell Dev. Biol. 9 681057 10.3389/fcell.2021.681057 34336833 PMC8316833

[7] Randow F. Youle R.J 2014 Self and nonself: how autophagy targets mitochondria and bacteria Cell Host Microbe 15 403 411 10.1016/j.chom.2014.03.012 24721569 PMC4238923

[8] Franklin T.G. Pruneda J.N 2021 Bacteria make surgical strikes on host ubiquitin signaling PLoS Pathog. 17 e1009341 10.1371/journal.ppat.1009341 33735219 PMC7971504

[9] Roberts C.G. Franklin T.G. Pruneda J.N 2023 Ubiquitin-targeted bacterial effectors: rule breakers of the ubiquitin system EMBO J. 42 e114318 10.15252/embj.2023114318 37555693 PMC10505922

[10] Yan F. Huang C. Wang X. Tan J. Cheng S. Wan M et al. 2020 Threonine ADP-Ribosylation of ubiquitin by a bacterial effector family blocks host ubiquitination Mol. Cell 78 641 652 10.1016/j.molcel.2020.03.016 32330457

[11] Bhogaraju S. Kalayil S. Liu Y. Bonn F. Colby T. Matic I. et al. 2016 Phosphoribosylation of Ubiquitin Promotes Serine Ubiquitination and Impairs Conventional Ubiquitination Cell 167 1636 1649 10.1016/j.cell.2016.11.019 27912065

[12] Qiu J. Sheedlo M.J. Yu K. Tan Y. Nakayasu E.S. Das C. et al. 2016 Ubiquitination independent of E1 and E2 enzymes by bacterial effectors Nature 533 120 124 10.1038/nature17657 27049943 PMC4905768

[13] Pruneda J.N. Smith F.D. Daurie A. Swaney D.L. Villén J. Scott J.D et al. 2014 E2~Ub conjugates regulate the kinase activity of Shigella effector OspG during pathogenesis EMBO J. 33 437 449 10.1002/embj.201386386 24446487 PMC3989626

[14] Guan H. Fu J. Yu T. Wang Z.-X. Gan N. Huang Y. et al. 2020 Molecular basis of ubiquitination catalyzed by the bacterial transglutaminase mavC Adv. Sci. (Weinh). 7 2000871 10.1002/advs.202000871 32596129 PMC7312448

[15] Szczesna M. Huang Y. Lacoursiere R.E. Bonini F. Pol V. Koc F. et al. 2024 Bacterial esterases reverse lipopolysaccharide ubiquitylation to block host immunity Cell Host Microbe 32 913 924 10.1016/j.chom.2024.04.012 38870903 PMC11271751

[16] Misaghi S. Balsara Z.R. Catic A. Spooner E. Ploegh H.L. Starnbach M.N 2006 Chlamydia trachomatis-derived deubiquitinating enzymes in mammalian cells during infection Mol. Microbiol. 61 142 150 10.1111/j.1365-2958.2006.05199.x 16824101

[17] Bohnsack R.N. Haas A.L 2003 Conservation in the mechanism of Nedd8 activation by the human AppBp1-Uba3 heterodimer J. Biol. Chem. 278 26823 26830 10.1074/jbc.M303177200 12740388

[18] Duda D.M. Borg L.A. Scott D.C. Hunt H.W. Hammel M. Schulman B.A 2008 Structural insights into NEDD8 activation of cullin-RING ligases: conformational control of conjugation Cell 134 995 1006 10.1016/j.cell.2008.07.022 18805092 PMC2628631

[19] Noguchi K. Okumura F. Takahashi N. Kataoka A. Kamiyama T. Todo S. et al. 2011 TRIM40 promotes neddylation of IKKγ and is downregulated in gastrointestinal cancers Carcinogenesis 32 995 1004 10.1093/carcin/bgr068 21474709

[20] Harper J.W. Schulman B.A 2021 Cullin-ring ubiquitin ligase regulatory circuits: a quarter century beyond the f-box hypothesis Annu. Rev. Biochem. 90 403 429 10.1146/annurev-biochem-090120-013613 33823649 PMC8217159

[21] Vijayasimha K. Dolan B.P 2021 The many potential fates of non-canonical protein substrates subject to NEDDylation Cells 10 2660 10.3390/cells10102660 34685640 PMC8534235

[22] Kayesh M.E.H. Kohara M. Tsukiyama-Kohara K 2023 Effects of neddylation on viral infection: an overview Arch. Virol. 169 6 10.1007/s00705-023-05930-3 38081982

[23] Marchès O. Ledger T.N. Boury M. Ohara M. Tu X. Goffaux F. et al. 2003 Enteropathogenic and enterohaemorrhagic Escherichia coli deliver a novel effector called Cif, which blocks cell cycle G2/M transition Mol. Microbiol. 50 1553 1567 10.1046/j.1365-2958.2003.03821.x 14651638

[24] Morikawa H. Kim M. Mimuro H. Punginelli C. Koyama T. Nagai S. et al. 2010 The bacterial effector Cif interferes with SCF ubiquitin ligase function by inhibiting deneddylation of Cullin1 Biochem. Biophys. Res. Commun. 401 268 274 10.1016/j.bbrc.2010.09.048 20850415

[25] Yao Q. Cui J. Zhu Y. Wang G. Hu L. Long C. et al. 2009 A bacterial type III effector family uses the papain-like hydrolytic activity to arrest the host cell cycle Proc. Natl. Acad. Sci. U.S.A. 106 3716 3721 10.1073/pnas.0900212106 19225106 PMC2656146

[26] Cui J. Yao Q. Li S. Ding X. Lu Q. Mao H. et al. 2010 Glutamine deamidation and dysfunction of ubiquitin/NEDD8 induced by a bacterial effector family Science 329 1215 1218 10.1126/science.1193844 20688984 PMC3031172

[27] Crow A. Hughes R.K. Taieb F. Oswald E. Banfield M.J 2012 The molecular basis of ubiquitin-like protein NEDD8 deamidation by the bacterial effector protein Cif Proc. Natl. Acad. Sci. U.S.A. 109 E1830 8 10.1073/pnas.1112107109 22691497 PMC3390873

[28] Jubelin G. Taieb F. Duda D.M. Hsu Y. Samba-Louaka A. Nobe R. et al. 2010 Pathogenic bacteria target NEDD8-conjugated cullins to hijack host-cell signaling pathways PLOS Pathog. 6 e1001128 10.1371/journal.ppat.1001128 20941356 PMC2947998

[29] Yao Q. Cui J. Wang J. Li T. Wan X. Luo T. et al. 2012 Structural mechanism of ubiquitin and NEDD8 deamidation catalyzed by bacterial effectors that induce macrophage-specific apoptosis Proc. Natl. Acad. Sci. U.S.A. 109 20395 20400 10.1073/pnas.1210831109 23175788 PMC3528514

[30] Toro T.B. Toth J.I. Petroski M.D 2013 The cyclomodulin cycle inhibiting factor (CIF) alters cullin neddylation dynamics J. Biol. Chem. 288 14716 14726 10.1074/jbc.M112.448258 23589306 PMC3663497

[31] Pruneda J.N. Durkin C.H. Geurink P.P. Ovaa H. Santhanam B. Holden D.W et al. 2016 The molecular basis for ubiquitin and ubiquitin-like specificities in bacterial effector proteases Mol. Cell 63 261 276 10.1016/j.molcel.2016.06.015 27425412 PMC4961225

[32] Le Negrate G. Krieg A. Faustin B. Loeffler M. Godzik A. Krajewski S. et al. 2008 ChlaDub1 of Chlamydia trachomatis suppresses NF-kappaB activation and inhibits IkappaBalpha ubiquitination and degradation Cell. Microbiol. 10 1879 1892 10.1111/j.1462-5822.2008.01178.x 18503636

[33] Fischer A. Harrison K.S. Ramirez Y. Auer D. Chowdhury S.R. Prusty B.K. et al. 2017 *Chlamydia trachomatis*-containing vacuole serves as deubiquitination platform to stabilize Mcl-1 and to interfere with host defense Elife 6 e21465 10.7554/eLife.21465 28347402 PMC5370187

[34] Wang X. Hybiske K. Stephens R.S 2018 Direct visualization of the expression and localization of chlamydial effector proteins within infected host cells Pathog. Dis. 76 fty011 10.1093/femspd/fty011 29390129 PMC6251622

[35] Pruneda J.N. Bastidas R.J. Bertsoulaki E. Swatek K.N. Santhanam B. Clague M.J. et al. 2018 A Chlamydia effector combining deubiquitination and acetylation activities induces Golgi fragmentation Nat. Microbiol. 3 1377 1384 10.1038/s41564-018-0271-y 30397340 PMC6319605

[36] Bastidas R.J. Kędzior M. Davidson R.K. Walsh S.C. Dolat L. Sixt B.S. et al. 2024 The acetylase activity of Cdu1 regulates bacterial exit from infected cells by protecting Chlamydia effectors from degradation Elife 12 10.7554/eLife.87386 PMC10942603 38358795

[37] Sharma M. Fuertes D. Perez-Gil J. Lois L.M 2021 SUMOylation in Phytopathogen Interactions: Balancing Invasion and Resistance Front. Cell Dev. Biol. 9 703795 10.3389/fcell.2021.703795 34485289 PMC8415633

[38] Ribet D. Hamon M. Gouin E. Nahori M.-A. Impens F. Neyret-Kahn H. et al. 2010 Listeria monocytogenes impairs SUMOylation for efficient infection Nature 464 1192 1195 10.1038/nature08963 20414307 PMC3627292

[39] Youssouf N. Recasens-Zorzo C. Molle V. Bossis G. Soubeyran P. Gannoun-Zaki L 2021 *Staphylococcus aureus* Decreases SUMOylation Host Response to Promote Intramacrophage Survival Int. J. Mol. Sci. 22 8108 10.3390/ijms22158108 34360873 PMC8347835

[40] Loison L. Huré M. Lefranc B. Leprince J. Bôle-Feysot C. Coëffier M. et al. 2025 *Staphylococcus warneri* dampens SUMOylation and promotes intestinal inflammation Gut Microbes 17 2446392 10.1080/19490976.2024.2446392 39819277 PMC12931719

[41] Sidik S.M. Salsman J. Dellaire G. Rohde J.R 2015 Shigella Infection Interferes with SUMOylation and Increases PML-NB Number PLOS ONE 10 e0122585 10.1371/journal.pone.0122585 25848798 PMC4388590

[42] Verma S. Mohapatra G. Ahmad S.M. Rana S. Jain S. Khalsa J.K. et al. 2015 Salmonella Engages Host MicroRNAs To Modulate SUMOylation: a New Arsenal for Intracellular Survival Mol. Cell. Biol. 35 2932 2946 10.1128/MCB.00397-15 26100020 PMC4525320

[43] Lapaquette P. Fritah S. Lhocine N. Andrieux A. Nigro G. Mounier J. et al. 2017 Shigella entry unveils a calcium/calpain-dependent mechanism for inhibiting sumoylation Elife 6 e27444 10.7554/eLife.27444 29231810 PMC5745084

[44] Jo K. Kim E.J. Yu H.J. Yun C.-H. Kim D.W 2017 Host Cell Nuclear Localization of Shigella flexneri Effector OspF Is Facilitated by SUMOylation J. Microbiol. Biotechnol. 27 610 615 10.4014/jmb.1611.11066 27994213

[45] Beyer A.R. Truchan H.K. May L.J. Walker N.J. Borjesson D.L. Carlyon J.A 2015 The Anaplasma phagocytophilum effector AmpA hijacks host cell SUMOylation Cell. Microbiol. 17 504 519 10.1111/cmi.12380 25308709 PMC4664186

[46] Truchan H.K. Cockburn C.L. May L.J. VieBrock L. Carlyon J.A 2016 Anaplasma phagocytophilum-Occupied Vacuole Interactions with the Host Cell Cytoskeleton Vet. Sci. 3 25 10.3390/vetsci3030025 29056733 PMC5606578

[47] Dunphy P.S. Luo T. McBride J.W 2014 Ehrlichia chaffeensis exploits host SUMOylation pathways to mediate effector-host interactions and promote intracellular survival Infect. Immun. 82 4154 4168 10.1128/IAI.01984-14 25047847 PMC4187855

[48] Dzimianski J.V. Scholte F.E.M. Bergeron É. Pegan S.D 2019 ISG15: It’s Complicated J. Mol. Biol. 431 4203 4216 10.1016/j.jmb.2019.03.013 30890331 PMC6746611

[49] Chiang C. Liu G. Gack M.U 2021 Viral Evasion of RIG-I-like receptor-mediated immunity through dysregulation of ubiquitination and ISGylation Viruses 13 182 10.3390/v13020182 33530371 PMC7910861

[50] Ali M.S. Tang Y.S. Lee H.H.Y. Baker S.C. Mok C.K.P 2025 ISG-15, beyond its functions in the cell: a mini review Cell. Mol. Life Sci. 82 289 10.1007/s00018-025-05705-w 40719862 PMC12304407

[51] Durfee L.A. Lyon N. Seo K. Huibregtse J.M 2010 The ISG15 conjugation system broadly targets newly synthesized proteins: implications for the antiviral function of ISG15 Mol. Cell 38 722 732 10.1016/j.molcel.2010.05.002 20542004 PMC2887317

[52] Thery F. Martina L. Asselman C. Zhang Y. Vessely M. Repo H. et al. 2021 Ring finger protein 213 assembles into a sensor for ISGylated proteins with antimicrobial activity Nat. Commun. 12 5772 10.1038/s41467-021-26061-w 34599178 PMC8486878

[53] Swaim C.D. Canadeo L.A. Monte K.J. Khanna S. Lenschow D.J. Huibregtse J.M 2020 modulation of extracellular ISG15 Signaling by Pathogens and Viral Effector Proteins Cell Rep. 31 107772 10.1016/j.celrep.2020.107772 32553163 PMC7297157

[54] Bogunovic D. Byun M. Durfee L.A. Abhyankar A. Sanal O. Mansouri D. et al. 2012 Mycobacterial disease and impaired IFN-γ immunity in humans with inherited ISG15 deficiency Science 337 1684 1688 10.1126/science.1224026 22859821 PMC3507439

[55] Radoshevich L. Impens F. Ribet D. Quereda J.J. Nam Tham T. Nahori M.-A. et al. 2015 ISG15 counteracts Listeria monocytogenes infection Elife 4 e06848 10.7554/eLife.06848 26259872 PMC4530601

[56] Zhang Y. Thery F. Wu N.C. Luhmann E.K. Dussurget O. Foecke M. et al. 2019 The in vivo ISGylome links ISG15 to metabolic pathways and autophagy upon Listeria monocytogenes infection Nat. Commun. 10 5383 10.1038/s41467-019-13393-x 31772204 PMC6879477

[57] Zhang Y. Ripley B. Ouyang W. Sturtz M. Upton E. Luhmann E. et al. ISG15-modification of the Arp2/3 complex restricts pathogen spread Cell Biology 10.1101/2022.12.27.522022

[58] Wu Y. Liu C. Tang C. Niragire B. Levy-Zauberman Y. Adapen C. et al. 2024 *Chlamydia* -driven ISG15 expression dampens the immune response of epithelial cells independently of ISGylation MBio 15 10.1128/mbio.02401-24 PMC11559041 39345209

[59] Kimmey J.M. Campbell J.A. Weiss L.A. Monte K.J. Lenschow D.J. Stallings C.L 2017 The impact of ISGylation during Mycobacterium tuberculosis infection in mice Microbes Infect 19 249 258 10.1016/j.micinf.2016.12.006 28087453 PMC5403610

[60] Cao S. Dou X. Zhang X. Fang Y. Yang Z. Jiang Y et al. 2021 Streptococcus pneumoniae autolysin LytA inhibits ISG15 and ISGylation through decreasing bacterial DNA abnormally accumulated in the cytoplasm of macrophages Mol. Immunol 140 87 96 10.1016/j.molimm.2021.09.011 34673375

[61] Arimoto K.-I. Löchte S. Stoner S.A. Burkart C. Zhang Y. Miyauchi S. et al. 2017 STAT2 is an essential adaptor in USP18-mediated suppression of type I interferon signaling Nat. Struct. Mol. Biol. 24 279 289 10.1038/nsmb.3378 28165510 PMC5365074

[62] Klionsky D.J. Schulman B.A 2014 Dynamic regulation of macroautophagy by distinctive ubiquitin-like proteins Nat. Struct. Mol. Biol. 21 336 345 10.1038/nsmb.2787 24699082 PMC4036234

[63] Randow F. MacMicking J.D. James L.C 2013 Cellular self-defense: how cell-autonomous immunity protects against pathogens Science 340 701 706 10.1126/science.1233028 23661752 PMC3863583

[64] Choy A. Dancourt J. Mugo B. O’Connor T.J. Isberg R.R. Melia T.J. et al. 2012 The Legionella effector RavZ inhibits host autophagy through irreversible Atg8 deconjugation Science 338 1072 1076 10.1126/science.1227026 23112293 PMC3682818

[65] Swatek K.N. Aumayr M. Pruneda J.N. Visser L.J. Berryman S. Kueck A.F. et al. 2018 Irreversible inactivation of ISG15 by a viral leader protease enables alternative infection detection strategies Proc. Natl. Acad. Sci. U.S.A. 115 2371 2376 10.1073/pnas.1710617115 29463763 PMC5877979

[66] Hermanns T. Kolek S. Uthoff M. De Heiden R.A. Mulder M.P.C. Baumann U et al. 2025 A family of bacterial Josephin-like deubiquitinases with an irreversible cleavage mode Mol. Cell 85 1202 1215 10.1016/j.molcel.2025.02.002 40037356

[67] Shi Y. Liu H. Ma K. Luo Z.-Q. Qiu J 2023 Legionella longbeachae effector protein RavZ inhibits autophagy and regulates phagosome ubiquitination during infection PLOS ONE 18 e0281587 10.1371/journal.pone.0281587 36758031 PMC9910735

[68] Sudhakar P. Jacomin A.-C. Hautefort I. Samavedam S. Fatemian K. Ari E. et al. 2019 Targeted interplay between bacterial pathogens and host autophagy Autophagy 15 1620 1633 10.1080/15548627.2019.1590519 30909843 PMC6693458

[69] Yu D. Yin Z. Jin Y. Zhou J. Ren H. Hu M. et al. 2016 Evolution of *bopA* Gene in *Burkholderia* : a case of convergent evolution as a mechanism for bacterial autophagy evasion . Biomed Res. Int. 2016 1 7 10.1155/2016/6745028 PMC5149610 28018913

[70] Joompa P. Ponnikorn S. Roytrakul S. Tungpradabkul S 2017 Investigation of host-pathogen interaction between *Burkholderia pseudomallei* and autophagy-related protein LC3 using hydrophobic chromatography-based technique Cell Biosci. 7 45 10.1186/s13578-017-0172-4 28852470 PMC5567900

[71] Gong L. Cullinane M. Treerat P. Ramm G. Prescott M. Adler B. et al. 2011 The Burkholderia pseudomallei type iii secretion system and bopa are required for evasion of lc3-associated phagocytosis PLOS ONE 6 e17852 10.1371/journal.pone.0017852 21412437 PMC3055895

[72] Aichem A. Sailer C. Ryu S. Catone N. Stankovic-Valentin N. Schmidtke G. et al. 2019 The ubiquitin-like modifier FAT10 interferes with SUMO activation Nat. Commun. 10 4452 10.1038/s41467-019-12430-z 31575873 PMC6773726

[73] Negi H. Ravichandran A. Dasgupta P. Reddy S. Das R 2024 Plasticity of the proteasome-targeting signal Fat10 enhances substrate degradation Elife 13 e91122 10.7554/eLife.91122 38984715 PMC11299979

[74] Mueller S. Bialas J. Ryu S. Catone N. Aichem A 2023 The ubiquitin-like modifier FAT10 covalently modifies HUWE1 and strengthens the interaction of AMBRA1 and HUWE1 PLOS ONE 18 e0290002 10.1371/journal.pone.0290002 37578983 PMC10424871

[75] Bialas J. Boehm A.N. Catone N. Aichem A. Groettrup M 2019 The ubiquitin-like modifier FAT10 stimulates the activity of deubiquitylating enzyme OTUB1 Journal of Biological Chemistry 294 4315 4330 10.1074/jbc.RA118.005406 30718280 PMC6433071

[76] Saxena K. Inholz K. Basler M. Aichem A 2024 FAT10 inhibits TRIM21 to down-regulate antiviral type-I interferon secretion Life Sci. Alliance 7 e202402786 10.26508/lsa.202402786 38977311 PMC11231494

[77] Lukasiak S. Schiller C. Oehlschlaeger P. Schmidtke G. Krause P. Legler D.F. et al. 2008 Proinflammatory cytokines cause FAT10 upregulation in cancers of liver and colon Oncogene 27 6068 6074 10.1038/onc.2008.201 18574467

[78] Spinnenhirn V. Farhan H. Basler M. Aichem A. Canaan A. Groettrup M 2014 The ubiquitin-like modifier FAT10 decorates autophagy-targeted Salmonella and contributes to Salmonella resistance in mice J. Cell. Sci. 127 4883 4893 10.1242/jcs.152371 25271057

[79] Millrine D. Peter J.J. Kulathu Y 2023 A guide to UFMylation, an emerging posttranslational modification FEBS J. 290 5040 5056 10.1111/febs.16730 36680403 PMC10952357

[80] Jing J. Yang F. Wang K. Cui M. Kong N. Wang S. et al. 2025 UFMylation of NLRP3 Prevents its autophagic degradation and facilitates inflammasome activation Adv. Sci. (Weinh). 12 2406786 10.1002/advs.202406786 39985286 PMC12005806

[81] Li Y.Y. Zhang G.Y. He J.P. Zhang D.D. Kong X.X. Yuan H.M. et al. 2017 Ufm1 inhibits LPS-induced endothelial cell inflammatory responses through the NF-κB signaling pathway Int. J. Mol. Med. 39 1119 1126 10.3892/ijmm.2017.2947 28393202 PMC5403479

[82] Balce D.R. Wang Y.-T. McAllaster M.R. Dunlap B.F. Orvedahl A. Hykes B.L. Jr et al. 2021 UFMylation inhibits the proinflammatory capacity of interferon-γ-activated macrophages Proc. Natl. Acad. Sci. U.S.A. 118 e2011763118 10.1073/pnas.2011763118 33372156 PMC7817147

[83] Jeng E.E. Bhadkamkar V. Ibe N.U. Gause H. Jiang L. Chan J et al. 2019 Systematic Identification of Host Cell Regulators of Legionella pneumophila Pathogenesis Using a Genome-wide CRISPR Screen Cell Host Microbe 26 551 563 10.1016/j.chom.2019.08.017 31540829 PMC6800164

[84] López-Jiménez A.T. Théry F. Wright K. Painter H. Hoffmeister S.T. Jarche L. et al. 2025 Proximity biotinylation at the host-*Shigella* interface reveals UFMylation as an antibacterial pathway bioRxiv 10.1101/2025.05.29.656827 40501833 PMC12154702

[85] Schubert A.F. Nguyen J.V. Franklin T.G. Geurink P.P. Roberts C.G. Sanderson D.J. et al. 2020 Identification and characterization of diverse OTU deubiquitinases in bacteria EMBO J. 39 e105127 10.15252/embj.2020105127 32567101 PMC7396840

[86] Roberts C.G. Kaur S. Ogden A.J. Divine M.E. Warren G.D. Kang D. et al. 2024 A functional screen for ubiquitin regulation identifies an E3 ligase secreted by *Pseudomonas aeruginosa* bioRxiv 10.1101/2024.09.18.613774 39345563 PMC11430079

